# Otosyphilis: A Rare Case of Unilateral Acute Sensorineural Hearing Loss

**DOI:** 10.7759/cureus.77338

**Published:** 2025-01-12

**Authors:** Shinji Iwata, Seitaro Murakawa, Naoya Nishida, Naohito Hato

**Affiliations:** 1 Otolaryngology-Head and Neck Surgery, Ehime University Graduate School of Medicine, Toon, JPN; 2 Otorhinolaryngology, Ehime Prefectural Niihama Hospital, Niihama, JPN

**Keywords:** neurosyphilis, sexually transmitted disease (std), sudden sensorineural hearing loss, syphilis, treponema pallidum

## Abstract

Otosyphilis is a rare cause of acute sensorineural hearing loss. This report presents a case of otosyphilis causing unilateral acute sensorineural hearing loss in a 50-year-old female. The initial symptoms included left-sided hearing loss, tinnitus, headache, and nausea, which persisted despite oral steroid therapy. Blood tests revealed positive syphilis serology and cerebrospinal fluid (CSF) analysis indicated neurosyphilis. Treatment with ampicillin (AMPC) and tapered steroid therapy improved the symptoms, except for residual impairment at a frequency of 1 kHz. This case highlights the significance of considering syphilis in the differential diagnosis of acute sensorineural hearing loss, thorough history taking, serological testing, and when indicated, CSF analysis to ensure timely diagnosis and management.

## Introduction

Syphilis, caused by *Treponema pallidum*, is a sexually transmitted disease with a wide range of localized and systemic manifestations. In Japan, the number of syphilis cases has increased since 2010, posing a public health concern [1]. Acute sensorineural hearing loss associated with syphilis typically presents with nonspecific findings, making diagnosis challenging [2]. This report presents a case of otosyphilis causing unilateral acute sensorineural hearing loss, which was treated with steroids and oral antibiotics.

## Case presentation

A previously healthy 50-year-old female patient was admitted to our hospital with left-sided hearing loss, tinnitus, headache, and nausea. Up to three months before presentation, the patient had multiple episodes of sexual intercourse with men who were not regular partners. She had experienced headaches and nausea in the past month without identifiable triggers. Two weeks before admission, she developed left-sided hearing loss and a sensation of ear fullness and visited a local otolaryngologist. She was diagnosed with acute sensorineural hearing loss and started on oral prednisolone (PSL) 30 mg/day; however, her symptoms did not improve. The patient was referred to our department.

**Figure 1:**
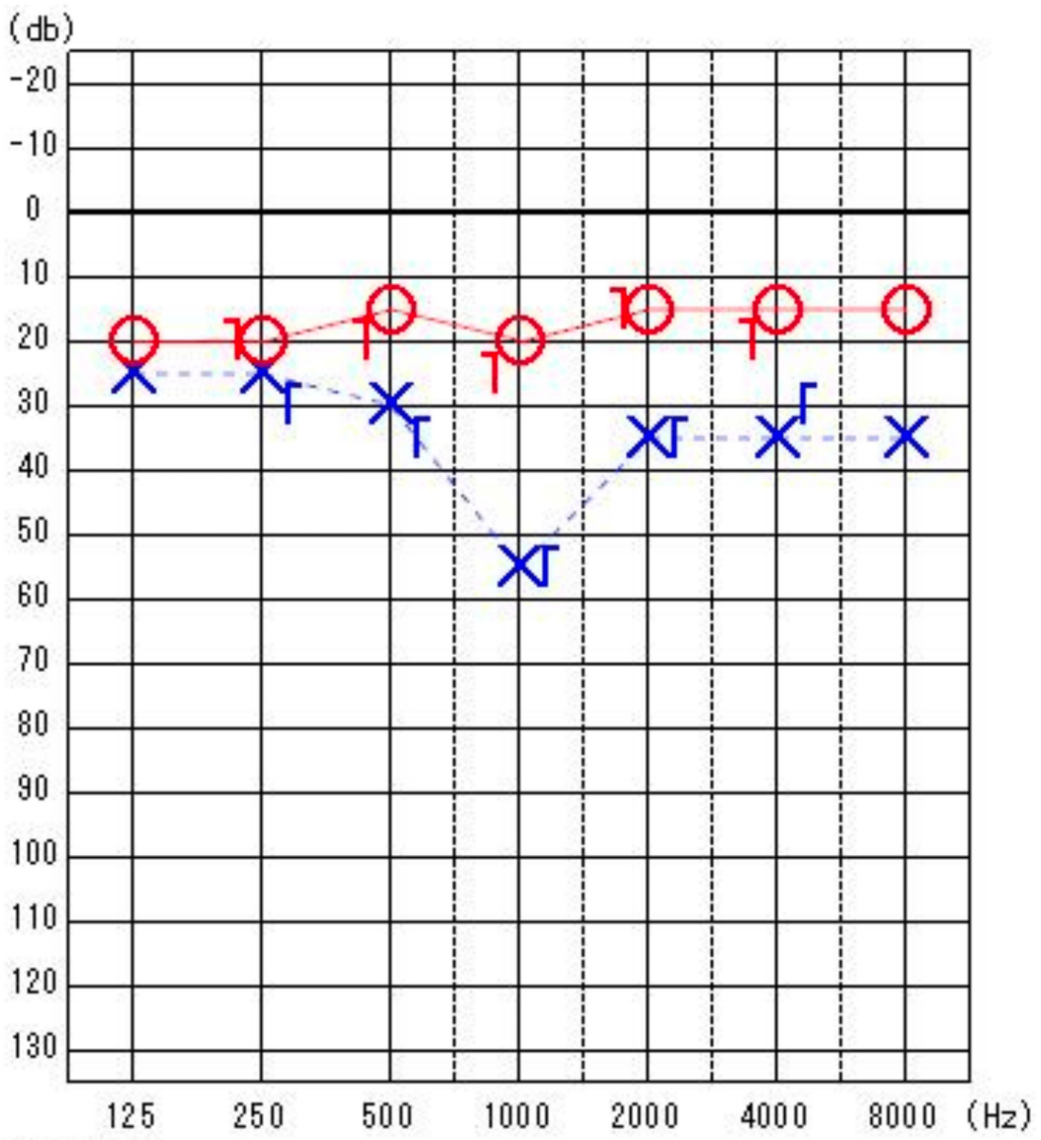
Pure-tone audiometry The findings indicated left sensorineural hearing loss centered around 1 kHz.

**Figure 2:**
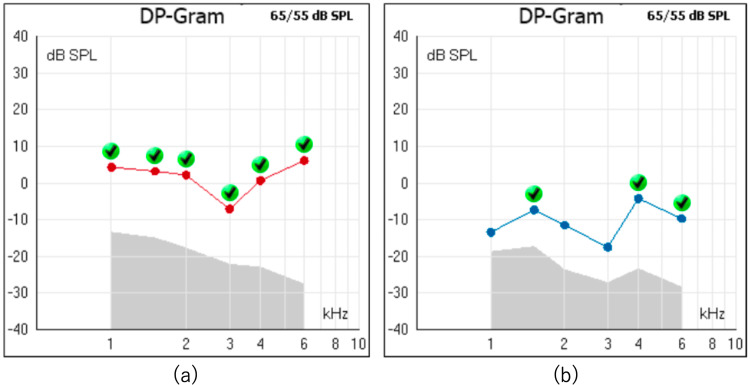
Distortion-product otoacoustic emissions (DPOAE) (a) Good response in the right ear; (b) Poor response at 1, 2, and 3 kHz in the left ear

**Figure 3:**
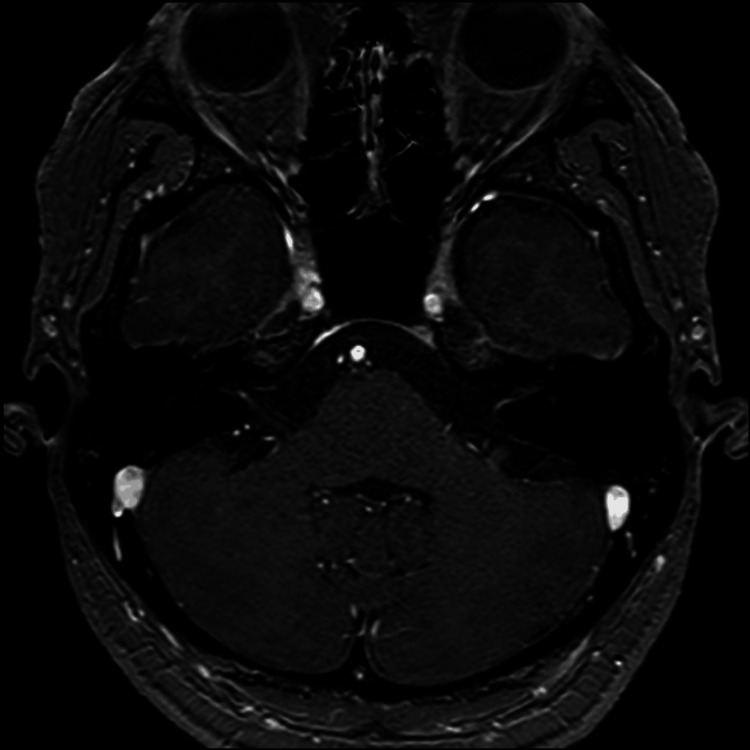
Contrast-enhanced MRI No obvious abnormal signals or contrast effects were observed in the internal auditory canal.

Physical examination revealed no abnormalities in the external auditory canal or tympanic membrane. The oral cavity and oropharynx were unremarkable, and cervical lymphadenopathy was not palpable. At her first visit, pure-tone audiometry revealed air conduction five-frequency average hearing levels of 17.0 dB in the right ear and 36.0 dB in the left ear, showing sensorineural hearing loss centered at 1 kHz (Figure 1). Distortion-product otoacoustic emissions (DPOAE) revealed poor responses at certain frequencies (Figure 2). Based on the diagnosis of left-sided acute sensorineural hearing loss, she was admitted for treatment, and blood tests were positive for both rapid plasma regain (RPR) and *Treponema pallidum* antibody (TPAb) (Table 1). Subsequently, cerebrospinal fluid (CSF) analysis and contrast-enhanced MRI were performed. The CSF analysis revealed an increased mononuclear cell count and elevated protein levels. Further testing yielded positive results for CSF fluorescent treponemal antibody absorption (FTA-ABS) and TPAb (Table 2). Contrast-enhanced MRI revealed no abnormal signals in the internal auditory canal (Figure 3).

**Table 1:** The patient's laboratory results WBC: white blood cells; RBC: red blood cells; AST: aspartate transaminase; ALT: alanine transaminase; ALP: alkaline phosphatase; RPR: rapid plasma regain; TPAb: *Treponema pallidum* antibody; HBs: hepatitis B surface antigen; HCV: hepatitis C virus; HBc-antibody: hepatitis B core antibody; HBs-antibody: hepatitis B surface antibody; HIV: human immunodeficiency virus

Test	Results	Reference range
Complete blood count		
WBC	13.4× 10^3^/μL	3.3-8.6 × 10^3^/μL
RBC	4.08 × 10^3^/μL	3.86-4.92 × 10^3^/μL
Hemoglobin	13.0 g/dL	11.6-14.8 g/dL
Hematocrit	38.9 %	35.1-44.4 %
Platelets	32.1 × 10^4^/μL	15.8-34.8 × 10^4^/μL
Liver function Tests		
AST	14 U/L	3-38 U/L
ALT	12 U/L	4-44 U/L
ALP	121 U/L	38-113 U/L
Total protein	7.2 g/dL	6.6-8.1 g/dL
C-reactive protein	0.113 mg/dL	0.00-0.40 mg/dL
Urea and electrolytes		
Sodium	140 mEq/L	136-148 mEq/L
Potassium	3.7 mEq/L	3.6-5.0 mEq/L
Chloride	105 mEq/L	98-108 mEq/L
Urea nitrogen	10.4 mg/dL	6.0-21.0 mg/dL
Creatinine	0.55 mg/dL	0.65-1.09 mg/dL
Infectious diseases		
RPR	28.4 R.U.	0.0-0.9 R.U.
TPAb	23.83 S/CO	<1.00 S/CO
HBs	≦0.02	0.00-0.05 IU/ml
HCV	0.09 S/CO	0.00-0.99 S/CO
HBc-antibody	0.10	0.0-1.0
HBs-antibody	<2.5	0.0-9.9
HIV-1, 2	<1.00 S/CO	0.0-0.9 S/CO

**Table 2:** Cerebrospinal fluid (CSF) test results The CSF analysis revealed an increased mononuclear cell count and elevated protein levels. Further testing yielded positive results for CSF FTA-ABS and TPAb. FTA-ABS: fluorescent treponemal antibody absorption; TPAb: *Treponema pallidum *antibodies

Test	Results	Reference range
Specific gravity	1.005	1.005-1.007
Cell count	222 /μL	0-5 /μL
Mononuclear	217 /μL (98%)	
Multinuclear	5 /μL (2%)	
Protein	114 mg/dL	10-40 mg/dL
Sugar	44 mg/dL	50-75 mg/dL
FTA-ABS	128	
TPAb	5120	

A dermatology consultation was requested for a full-body examination, which revealed no lymphadenopathy in the cervical, axillary, or inguinal regions and no rashes or ulcerative lesions in the external genital area. The speech discrimination scores were 95% bilaterally, and auditory brainstem response (ABR) testing indicated normal response thresholds and I-V wave latencies within the normal limits. No spontaneous, gaze-evoked, or positional nystagmus was observed. Additionally, the eye tracking test (ETT), optokinetic nystagmus (OKN), and optokinetic pattern test (OKP) results were within normal limits.

**Figure 4:**
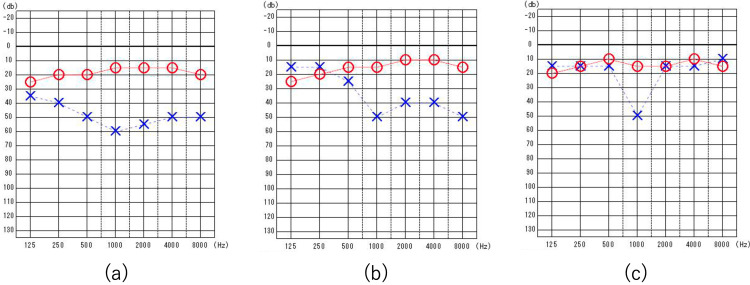
Pure-tone audiometry (a) Shortly before the start of treatment; compared with the initial hearing test, an increase in the hearing threshold of the left ear was observed. (b) One month after the start of treatment; a decrease in the low-frequency threshold was observed. (c) Two months after the start of treatment; A decrease in the high-frequency threshold was observed. No change was noted in the 1 kHz threshold.

Treatment was initiated on the third day after the completion of the various tests. Hearing tests performed immediately before treatment initiation revealed a further increase in the left hearing threshold (Figure 4a). The treatment consisted of a gradually tapered PSL dose starting at 60 mg and oral ampicillin (AMPC) at 1500 mg/day. The patient’s complaints of headache and nausea resolved within a few days of treatment initiation. After one month of treatment, an improvement in the hearing threshold at low frequencies in the left ear was observed, and her hearing loss and sensation of ear fullness resolved (Figure 4b). After two months of treatment, a decrease in the high-frequency hearing threshold in the left ear was noted, although the hearing level at 1 kHz remained unchanged (Figure 4c). The AMPC regimen was completed after three months. Quantitative RPR testing revealed a gradual decline, reaching less than one-fourth of the pre-treatment level six months after treatment initiation. Audiological tests did not reveal further changes after two months of treatment, and DPOAE responses at 1 kHz remained absent. Further quantitative RPR testing was scheduled at 12 and 24 months posttreatment, along with continued audiological follow-up.

## Discussion

Otosyphilis is a rare complication of neurosyphilis, a condition in which the central nervous system is infected with Treponema pallidum. After the initial infection, neurosyphilis is known to infiltrate the central nervous system in 25%-60% of cases, often asymptomatically, although 5% of cases may develop meningitis, cranial neuritis, ocular syphilis, or meningovascular syphilis [3]. In this case report, the patient tested positive for syphilis in blood tests. Given her symptoms of headache and nausea, CSF analysis was conducted, which revealed findings suggestive of syphilis infection. Although no skin symptoms were identified based on her medical history and test results, she was diagnosed with neurosyphilis secondary to latent syphilis and was treated accordingly.

Treponema pallidum haemagglutination (TPHA) and RPR in blood tests, as well as FTA-ABS, venereal disease research laboratory (VDRL), cell counts, and protein levels in CSF analysis are considered useful for the diagnosis of neurosyphilis [4]. In this case, the blood tests were positive for syphilis and FTA-ABS, and there was an increase in the CSF cell count and protein levels, which was consistent with the diagnosis (Table 3). The VDRL test is recommended for the definitive diagnosis of neurosyphilis because of its high specificity [5,6]. However, because VDRL testing is unavailable in Japan, the diagnosis must be based on a comprehensive assessment of CSF and neurological findings.

**Table 3:** Clinical tests for neurosyphilis and the results of this case Note: Partially cited from reference [4], with the results of this case added. In this case, the blood tests were positive for syphilis and FTA-ABS, and there was an increase in the CSF cell count and protein levels, which was consistent with the diagnosis. TPHA: *Treponema pallidum* haemagglutination; RPR: rapid plasma regain; FTA-ABS: fluorescent treponemal antibody absorption; CSF: cerebrospinal fluid; VDRL: venereal disease research laboratory

Particulars	Sensitivity		Specificity		This case
	Test	Early stage (%)	Late stage (%)	Late stage (%)	
Serologic tests	TPHA	100	96	60	Positive
	RPR	100	50-75	90	Positive
CSF tests	FTA-ABS	100	99	50-70	Positive
	VDRL	75	30-70	100	-
	White-cell count (5-10/mm^3^)	100	95	97	Positive (222/μL)
	Protein (>45 mg/dL)	90	95	<50	Positive (114mg/dL)

Magnetic resonance imaging studies have reported enhancement effects in the inner ear and meninges [7], although no significant abnormalities were noted in this case.

Hearing loss in neurosyphilis can follow two pathways: progression from meningitis to cranial neuritis of the seventh and eighth cranial nerves or hematogenous spread to the inner ear, causing inner ear-type hearing loss [7,8]. In the present case, the CSF findings suggested meningitis progressing to the eighth cranial nerve neuritis; however, the poor response in DPOAE testing also raised the possibility of inner ear-type hearing loss. Otosyphilis can manifest with symptoms other than hearing loss, such as vertigo and tinnitus. The improvement rates for vertigo (86%) and tinnitus (85%) have been reported to be higher than those for hearing loss (31%) [9]. Prognostic factors for favorable hearing recovery include onset within five years, age < 60 years, and fluctuating hearing loss. The patient met the criteria for age and duration. Although bilateral hearing loss is common, asymmetric or unilateral hearing loss has also been reported [2,10]. In the present case, only left-sided hearing loss was observed during follow-up, with no hearing impairment on the right side.

The treatment of neurosyphilis involves the use of antibiotics and corticosteroids. Antibiotic therapy typically involves intravenous penicillin G (PCG) at 18-24 million units/day for 10-14 days [11,12]. However, in this case, the patient did not wish to receive two weeks of intravenous therapy. There are reports in the literature of successful outcomes using intramuscular penicillin or oral antibacterial agents [13,14]. In this case, we opted for oral antibiotic therapy. Jarisch-Herxheimer reactions can occur within 24 hours of initiating antibiotic therapy. This reaction is caused by the destruction of *Treponema pallidum* and can lead to symptoms such as fever, headache, myalgia, and rash, which are usually self-limiting and managed with symptomatic treatment [15]. However, this reaction did not occur in the present case, although close monitoring after treatment initiation is essential.

Nevertheless, the role of corticosteroids in the treatment of neurosyphilis remains unclear. Histopathological studies of the temporal bones in otosyphilis have revealed endolymphatic hydrops caused by obstruction of the endolymphatic duct by microgranulomas. Steroid therapy can have anti-inflammatory and hydrops-relieving effects, thus supporting its use [16]. In the present case, corticosteroids were administered along with antibiotics, resulting in favorable outcomes. Although the thresholds for frequencies other than 1 kHz improved with treatment, suggesting suppression of syphilitic disease activity, DPOAE responses at 1 kHz remained absent six months after treatment, indicating irreversible damage at this frequency.

The evaluation of neurosyphilis treatment efficacy involves confirming symptom improvement and normalizing CSF findings [3]. However, a study has shown that if serum RPR titers decline appropriately (i.e., fourfold within 12 months), normalization of CSF findings can be anticipated, and repeat CSF testing may not be necessary [17]. In this case, although the 1 kHz hearing threshold did not improve, other frequencies showed improvement, the subjective symptoms resolved, and the serum RPR titers declined. Therefore, repeated CSF tests were not performed.

## Conclusions

Otosyphilis is a rare cause of acute sensorineural hearing loss; however, syphilitic infections should be considered in the differential diagnosis. It typically presents as general inner ear-related hearing loss; it often lacks specific findings, making it prone to being overlooked. A thorough medical history and blood tests are essential, and if findings suggestive of neurosyphilis are present, CSF analysis should be performed to ensure timely and appropriate treatment.
